# Novel Concepts for Intrauterine Device Placement at Caesarean Delivery: Description of Technique and Video Recording

**DOI:** 10.1155/2023/4410984

**Published:** 2023-05-05

**Authors:** G. Justus Hofmeyr, Kyungu M. Kime

**Affiliations:** ^1^Department of Obstetrics and Gynaecology, University of Botswana, Gaborone, Botswana; ^2^Effective Care Research Unit, Universities of the Witwatersrand, Fort Hare and Walter Sisulu and Eastern Cape Department of Health, East London, South Africa

## Abstract

The International Federation of Gynaecology and Obstetrics recommend digital insertion of the copper intrauterine device (IUD) during caesarean delivery and note the risk of thread inclusion in the uterotomy closure and nonvisibility of threads at follow-up. We describe a novel method of inserting the IUD with the insertion straw and directing the lower end of the straw through the cervix for retrieval after the operation, to protect and ensure alignment of the threads. We also describe a simple method of lengthening one thread with part of the other thread, to avoid risks associated with braided suture extensions.

## 1. Introduction

Access to effective postpartum contraception reduces unintended pregnancy and maternal and perinatal mortality. Copper intrauterine device (IUD) placement at caesarean delivery is promoted by the World Health Organization, the International Federation of Gynaecology and Obstetrics (FIGO), and the American College of Obstetricians and Gynaecologists, but is not yet widely used. Only 31% of French Obstetrician/Gynaecologists recently surveyed knew that the IUD could be placed at caesarean delivery, and 10% used the method [1]. In a meta-analysis of 12 reports from low-middle-income countries, continuation of IUD use at 6 months after postpartum insertion was 87%, but this was not broken down by mode of delivery [2].

A frameless copper IUD has been developed specifically for placement at caesarean delivery [3]. The anchoring knot is placed below the serosa of the uterus and fixed in place with a very thin absorbable suture. Expulsion has been found to be less frequent (1.4%) than with the copper T 380A framed IUD (11.4%) [4]. Most clinicians have used standard framed IUDs such as the copper T 380A for placement at caesarean delivery. In the absence of manufacturer instructions for “off-label' placement of the IUD at caesarean delivery, various improvised methods have been developed over the last 50 years. These include adding absorbable sutures to lengthen the threads, as well as methods of anchoring the IUD at the uterine fundus. FIGO recommends digital insertion of the IUD held between two fingers and notes the risk of string inclusion in the uterotomy closure and the nonvisibility of threads at follow-up [5]. Another method described is the insertion of the IUD in the applicator with arms out, external stabilisation of the uterus to hold the IUD in place, removing the applicator, and directing the threads into the cervix using ring forceps [6].

An important limitation of IUD insertion at caesarean delivery is the failure of the threads to become visible through the cervix.

Missing threads following postplacental IUD insertion were reported in 48% of cases at 45 to 90 days, and 34% at 6–9 months, being 6*x* more common with placement at caesarean delivery than after vaginal birth. [7].

In a Brazilian study of IUD placement at caesarean delivery, at 6 weeks and 6 months, continued use was 92% and 72% and satisfaction was 85% and 76%, respectively. Threads were nonvisible in 71% at 6 weeks [8]. For IUDs with long threads (for example, the Nova-T), the threads may be trimmed to about 20 cm from the top of the IUD prior to insertion. For models such as the copper T 380A IUD which is supplied with threads 14.5 cm measured from the top of the IUD, extension of the threads using absorbable sutures has been described. Disadvantages are that unless the absorbable thread is used to pull down the original thread before it is absorbed, it may be absorbed with the original threads still within the uterine cavity; and if a braided suture such as braided polyglycolic acid is used, it may act as a wick for ascending infections, as was the case with the Dalcon Shield IUD.

We describe two novel innovations for IUD placement at caesarean delivery designed to stabilise the IUD at the fundus during surgery, protect the threads from inclusion in the hysterotomy closure, and optimize the descent of the threads through the cervix.

## 2. Description of the Techniques

See the video for a demonstration of the technique https://www.youtube.com/watch?v=9qr5Uqxe61U. The IUD is placed after delivery of the placenta and suturing of the angles of the hysterotomy. The insertion straw with the plastic collar and plunger removed is used to insert the IUD (with side arms free) into the fundus of the uterus, guided digitally. The lower end of the straw is passed through the cervix and the straw is left in place to stabilise the IUD and protect the threads from inadvertent snaring in the uterotomy closure. The uterotomy is closed (we use the interrupted reverse figure of eight sutures excluding the decidua). At the end of the operation, the straw is retrieved transvaginally.

**Figure 1:**
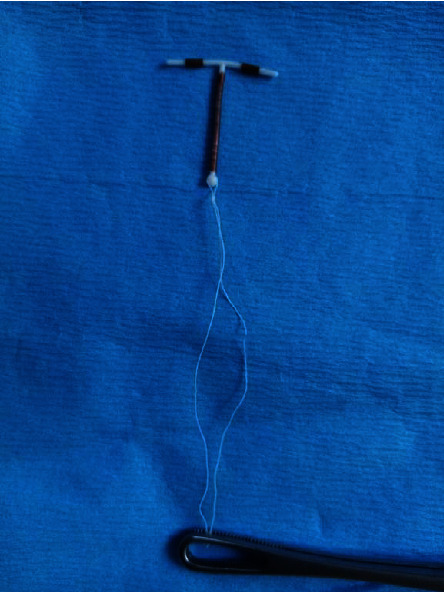
Thread lengthening: we hold the tips of both threads with a swab-holding or artery forceps and cut one string 5-6 cm from the forceps.

**Figure 2:**
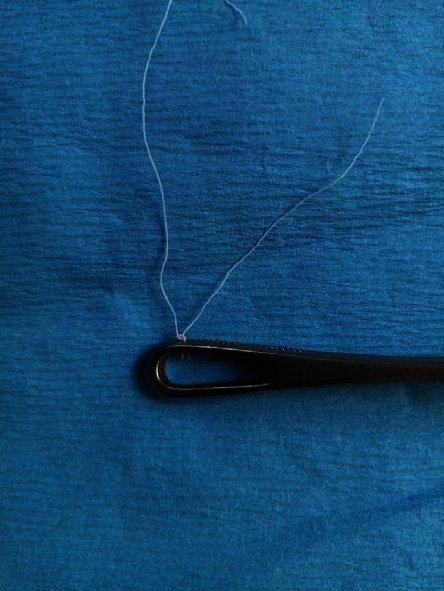
We tie with a simple reef knot.

**Figure 3:**
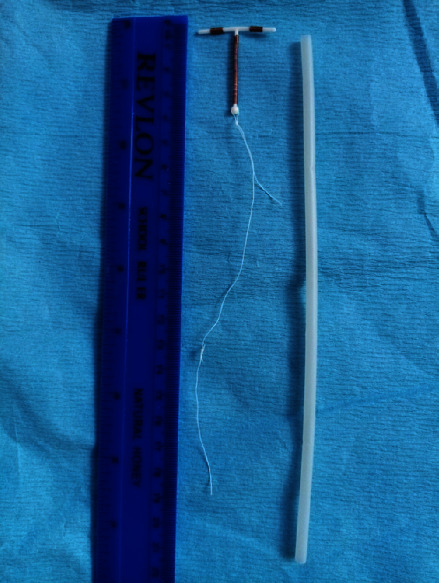
We pull the knot tight: one thread is lengthened from 14.5 cm to about 19.5 cm from the top of the IUD.

**Figure 4:**
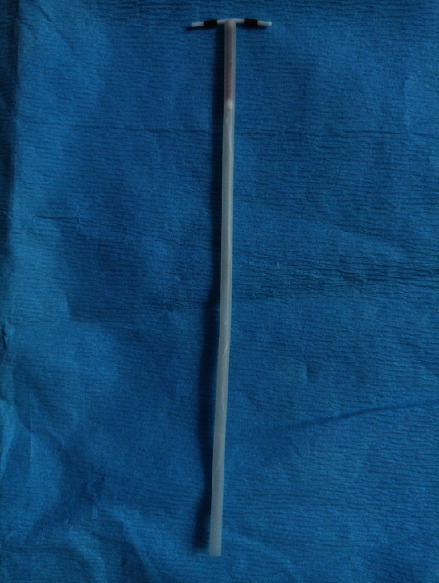
We replace the IUD in the straw for insertion.

The novel innovation we use for thread lengthening is to lengthen one thread of the IUD using part of the other thread. This is cost-free and avoids the use of surgical suture material which may absorb prematurely or act as a wick for ascending infection. We clamp the tips of both threads with a swab-holding or artery forceps; cut one thread 5-6 cm from the clamped point (Figure 1); tie the threads with a simple reef knot (Figure 2); pull the knot tight (Figure 3); and reinsert into the straw for insertion (Figure 4). As opposed to thread extensions with extraneous suture material, the single reef knot in the fine monofilament thread provided with the device is unlikely to cause significant risk.

Early experience with the delayed removal of insertion straw technique is that the IUD is consistently positioned within 15 mm of the uterine fundus on ultrasound examination at 48 hours.

## 3. Discussion

With the increasing use of IUD insertion at caesarean delivery globally, it is important that the optimal techniques for insertion be determined. Improvised techniques need to be made public so that robust trials comparing alternative methods can be mounted to determine which have the optimal results in terms of ease of use, the persistence of correct placement, and visibility of threads for confirming the presence of the IUD and removal.

In our experience, some women when counselled on the options would prefer their use of the IUD to be confidential and not detectable by their partner. We recommend that this issue be routinely discussed, including counselling on the disadvantages of not being able to confirm the presence of the IUD clinically and more difficult removal when threads are not visible. If requested, the threads may be cut short or purposefully coiled up within the uterine cavity, or tied in a small loop to facilitate removal.

After preparing this manuscript, we identified a recent publication describing a similar method of IUD insertion [9]. In a prospective nonrandomized study, failed insertion and uterine perforation were less common with insertion during caesarean delivery (0% each) than with interval insertion at 6 weeks (6% and 5%, respectively) while nonvisible threads were more common (13% versus 4% for interval insertion).

## Data Availability

Data sharing are not applicable as no new data are generated.

## Additional Points

Novel techniques using thread extension and insertion straw protection of threads for IUD insertion at caesarean delivery are illustrated by video recording.
